# Association of early life stress and cognitive performance in patients with schizophrenia and healthy controls

**DOI:** 10.1016/j.scog.2023.100280

**Published:** 2023-02-11

**Authors:** Fanny Senner, Thomas Schneider-Axmann, Lalit Kaurani, Jörg Zimmermann, Jens Wiltfang, Martin von Hagen, Thomas Vogl, Carsten Spitzer, Simon Senner, Eva C. Schulte, Max Schmauß, Sabrina K. Schaupp, Jens Reimer, Daniela Reich-Erkelenz, Sergi Papiol, Mojtaba Oraki Kohshour, Fabian U. Lang, Carsten Konrad, Sophie-Kathrin Kirchner, Janos L. Kalman, Georg Juckel, Maria Heilbronner, Urs Heilbronner, Christian Figge, Ruth E. Eyl, Detlef Dietrich, Monika Budde, Ion-George Angelescu, Kristina Adorjan, Andrea Schmitt, Andre Fischer, Peter Falkai, Thomas G. Schulze

**Affiliations:** a Department of Psychiatry and Psychotherapy, University Hospital, LMU Munich, Munich 80336, Germany; b Institute of Psychiatric Phenomics and Genomics (IPPG), University Hospital, LMU Munich, Munich 80336, Germany; c German Center of Neurodegenerative Diseases (DZNE), Göttingen 37075, Germany; d Psychiatrieverbund Oldenburger Land gGmbH, Karl-Jaspers-Klinik, Bad Zwischenahn 26160, Germany; e Department of Psychiatry and Psychotherapy, University Medical Center Göttingen, Göttingen 37075, Germany; f Neurosciences and Signaling Group, Institute of Biomedicine (iBiMED), Department of Medical Sciences, University of Aveiro, Aveiro, Portugal; g Clinic for Psychiatry and Psychotherapy, Clinical Center Werra-Meißner, Eschwege 37269, Germany; h Department of Psychosomatic Medicine and Psychotherapy, University Medical Center Rostock, Rostock 18147, Germany; i Center for Psychiatry Reichenau, Academic Hospital University of Konstanz, Konstanz 78479, Germany; j Department of Psychiatry and Psychotherapy, Bezirkskrankenhaus Augsburg, Augsburg 86156, Germany; k Department of Psychiatry and Psychotherapy, University Medical Center Hamburg-Eppendorf, Hamburg 20246, Germany; l Department of Immunology, Faculty of Medicine, Ahvaz Jundishapur University of Medical Sciences, Ahvaz, Iran; m Department of Psychiatry II, Ulm University, Bezirkskrankenhaus Günzburg, Günzburg, 89312, Germany; n Department of Psychiatry and Psychotherapy, Agaplesion Diakonieklinikum, Rotenburg 27356, Germany; o Department of Psychiatry, Ruhr University Bochum, LWL University Hospital, Bochum 44791, Germany; p Karl-Jaspers Clinic, European Medical School Oldenburg-Groningen, Oldenburg 26160, Germany; q Stuttgart Cancer Center –Tumorzentrum Eva Mayr-Stihl, Klinikum Stuttgart, Stuttgart 70174, Germany; r AMEOS Clinical Center Hildesheim, Hildesheim 31135, Germany; s Department of Psychiatry and Psychotherapy, Mental Health Institute Berlin, Berlin 14050, Germany; t Laboratory of Neuroscience (LIM27), Institute of Psychiatry, University of Sao Paulo, Sao Paulo, Brazil; u Cluster of Excellence “Multiscale Bioimaging: from Molecular Machines to Networks of Excitable Cells” (MBExC), University of Göttingen, Göttingen, Germany; v Department of Psychiatry and Behavorial Sciences, SUNY Upstate Medical University, Syracuse, 54, NY, USA; w Department of Psychiatry and Behavioral Sciences, Johns Hopkins University School of Medicine, Baltimore, MD, USA

**Keywords:** Schizophrenia, Healthy controls, Early life stress, Cognitive dysfunction

## Abstract

As core symptoms of schizophrenia, cognitive deficits contribute substantially to poor outcomes. Early life stress (ELS) can negatively affect cognition in patients with schizophrenia and healthy controls, but the exact nature of the mediating factors is unclear. Therefore, we investigated how ELS, education, and symptom burden are related to cognitive performance.

The sample comprised 215 patients with schizophrenia (age, 42.9 ± 12.0 years; 66.0 % male) and 197 healthy controls (age, 38.5 ± 16.4 years; 39.3 % male) from the PsyCourse Study. ELS was assessed with the Childhood Trauma Screener (CTS). We used analyses of covariance and correlation analyses to investigate the association of total ELS load and ELS subtypes with cognitive performance.

ELS was reported by 52.1 % of patients and 24.9 % of controls. Independent of ELS, cognitive performance on neuropsychological tests was lower in patients than controls (*p* < 0.001). ELS load was more closely associated with neurocognitive deficits (cognitive composite score) in controls (*r* = −0.305, *p* < 0.001) than in patients (*r* = −0.163, *p* = 0.033). Moreover, the higher the ELS load, the more cognitive deficits were found in controls (*r* = −0.200, *p* = 0.006), while in patients, this correlation was not significant after adjusting for PANSS.

ELS load was more strongly associated with cognitive deficits in healthy controls than in patients. In patients, disease-related positive and negative symptoms may mask the effects of ELS-related cognitive deficits. ELS subtypes were associated with impairments in various cognitive domains. Cognitive deficits appear to be mediated through higher symptom burden and lower educational level.

## Highlights

• Early life stress is often reported by patients with schizophrenia (52.1%) and healthy controls (24.9%).
• Early life stress is more strongly associated with neurocognitive deficits in controls than in patients with schizophrenia.
• The higher the early life stress load, the more symptoms and cognitive deficits are found.
• Disease-related symptoms may mask the effects of early life stress-related cognitive deficits in patients with schizophrenia.
• Cognitive deficits appear to be mediated through higher symptom burden and lower educational level.

## 1 Introduction

Early life stress (ELS) is a risk factor for mental illness, including psychoses (Aldinger and Schulze, 2017; Popovic et al., 2019; Schmitt et al., 2014; Varese et al., 2012). In the general population, ELS prevalences range from 3.8 % for sexual abuse to 20.9 % for physical abuse (McLaughlin et al., 2017).

In schizophrenia, ELS in general is known to negatively affect social cognition, working memory, executive function, verbal memory, and attention (Aas et al., 2012; Dauvermann and Donohoe, 2018; Kilian et al., 2018; Li et al., 2017; McCabe et al., 2012; Shannon et al., 2011). Cognitive deficits occur in 90 % of patients with schizophrenia, develop early, and have high longitudinal trait stability (Burdick et al., 2006; Douglas et al., 2018; Fioravanti et al., 2012; Fuller et al., 2002; Heinrichs and Zakzanis, 1998; Nakagome, 2017; Saykin et al., 1994). They greatly affect patients' lives (Carrión et al., 2011; Green, 1996; Green et al., 2012; Pascal de Raykeer et al., 2019): Only 20 % of patients work in the primary labor market, and only 30 % sustain a stable relationship over time (Häfner and an der Heiden, 2007). And also healthy individuals with ELS suffer from impaired neurocognition (Bücker et al., 2013; Dauvermann and Donohoe, 2018; Green et al., 2015; Poletti et al., 2017; Sideli et al., 2014; van Os et al., 2017). The association between ELS and cognitive function in schizophrenia shows a high variability (Dauvermann and Donohoe, 2018). In patients with psychosis and people at ultra-high risk for psychosis, ELS predicts early onset, worse social functioning, and unfavorable disease course (Yung et al., 2015). Robust data are lacking on associations between ELS subtypes and cognitive domains in patients with schizophrenia and healthy individuals. Aas et al. found that physical abuse, sexual abuse and physical neglect were significantly associated with reduced scores on working memory and executive function scales and verbal and performance tasks from the Wechsler Abbreviated Scale of Intelligence (Aas et al., 2012). There is little research evidence on mediating factors, such as symptom severity and education. A systematic review showed that these factors are not considered consistently (Dauvermann and Donohoe, 2018). Rahme et al. found that cognitive deficits did not mediate the association between ELS and psychotic symptoms (Rahme et al., 2023). Treatment of choice for cognitive deficits is neuropsychological training (Mororó et al., 2022); while other available treatments, e.g., antipsychotics and psychotherapy are little effective (Goff et al., 2011). A better understanding of the relationship between ELS and cognitive deficits could reduce possible therapy resistance and allow to address patients more individually. Also, effective prevention of ELS could help minimizing cognitive deficits a priori. The comparison between patients and healthy individuals can provide important insights into resilience, coping strategies, and disease susceptibility – beyond biological stress pathway models (Agorastos et al., 2019; Ruby et al., 2014).

In our study, we want to address the research question whether an association of ELS load and ELS subtypes with cognitive performance in patients with schizophrenia and healthy controls exists and whether the effect is mediated through other factors, such as education and symptom severity.

## 2 Methods

### 2.1 Study population

Study data were obtained in the multicenter, longitudinal, naturalistic, transdiagnostic *PsyCourse* Study, which was conducted in Germany and Austria (www.PsyCourse.de) between 2012 and 2019. This project aims on identifying clinical, neurobiological, molecular genetic signatures of the course of major psychiatric disorders. We used version 3.1 data (release 09/2018) (Budde et al., 2019) and included 215 patients with schizophrenia (142 men, 73 women) and 197 healthy controls (76 men, 121 women) who completed the Childhood Trauma Screener (CTS) (Grabe et al., 2012). Diagnoses were assessed with parts of the Structured Clinical Interview for DSM-IV (Wittchen et al., 1997). Healthy controls were assessed with the Mini-International Neuropsychiatric Interview for DSM-V (Sheehan et al., 1998).

The study was approved by the local ethics committee (Project number 17-13) and performed in accordance with the Declaration of Helsinki. All participants gave written informed consent.

### 2.2 Phenotypic data

Sociodemographic data comprised age, sex, marital and partnership status, number of children and siblings, living arrangement, education (main school or no degree/secondary school/high school), employment, and work absences. We also evaluated diagnosis, disease duration, psychiatric family history, treatment setting (outpatient/daypatient/inpatient), suicidal ideation, alcohol consumption, lifetime use of illicit drugs, current medication (number of antipsychotics, antidepressants, mood stabilizers, and tranquilizers), ELS, psychopathology, neurocognitive performance, and functioning. The *PsyCourse Codebook* (“Codebook PsyCourse,” n.d.) provides detailed information on phenotypic variables. Fixed data were taken from the baseline visit, and variable data, from visit 3 (month 12), as the CTS was assessed at this timepoint.

### 2.3 ELS

ELS was assessed at visit 3 with the CTS, a self-rated, five-point scale that assesses the five recognized types of childhood trauma (emotional and physical neglect and emotional, physical, and sexual abuse) (Bernstein et al., 2003; Grabe et al., 2012). Participants were classified into ELS and no-ELS groups according to validated threshold values (Glaesmer et al., 2013). The five ELS subtypes were analyzed separately (emotional neglect [CTS1, threshold ≥4], physical abuse [CTS2, threshold ≥3], emotional abuse [CTS3, threshold ≥3], sexual abuse [CTS4, threshold ≥2], and physical neglect [CTS5, threshold ≥4]). To assess the effect of ELS load on dependent variables, we built an ELS score from the five threshold values: 2 ∗ (CTS1-1) + 3 ∗ (CTS2-1) + 3 ∗ (CTS3-1) + 4 ∗ (CTS4-1) + 2 ∗ (CTS5-1).

### 2.4 Neurocognition

Neuropsychological testing was performed by raters trained in standardized neurocognitive assessment. We included data from visit 3, evaluated executive function, short-term and working memory, psychomotor speed, and learning and memory with the Trail Making Test (TMT), Verbal Digit Span (VDS), Digit Symbol Test (DST), and Verbal Learning Memory Test (VLMT) (Supplementary Material).

#### 2.4.1 Cognitive composite score

By multiplying negative scores by −1, if necessary, we created a positive cognitive composite score (CCS) from neuropsychological scores. Next, we calculated z-scores to create variables of comparable magnitude and summed TMT, VDS, DST, and VLMT z-scores with equal weighting (Hasan et al., 2016). Last, we calculated the composite score itself as a z-score.

### 2.5 Psychopathology and level of functioning

Severity of schizophrenia symptoms was evaluated with the Positive and Negative Syndrome Scale (PANSS) total score (Kay et al., 1987); severity of depressive symptoms, with the clinician-rated Inventory of Depressive Symptomatology (IDS-C_30_) (Drieling et al., 2007) and the self-rated Beck Depression Inventory II (BDI-II) (Kühner et al., 2007); severity of illness, with the Clinical Global Impression Scale (CGI) (Busner and Targum, 2007); level of functioning, with the Global Assessment of Functioning (GAF) (Aas, 2011). The respective data were taken from visit 3.

### 2.6 Statistical analysis

The independent variables were ELS (yes/no) and ELS load, and dependent variables were sociodemographic and clinical data and neuropsychological test results, including CCS.

To account for differences in analyses of clinical variables, we performed all analyses separately for patients and controls with two-tailed tests. We tested deviation from normal distribution of all dependent variables with Kolmogorov-Smirnov tests, and variance homogeneity, with Levene's tests.

In initial descriptive analyses, we compared demographic variables between ELS and no-ELS groups by analysis of variance (ANOVA) or Pearson's Chi-Square tests. We used Breslow-Day test to analyze whether odds ratios were homogenous between female and male patients and applied Cochran's test for conditional independence between the sexes. As preliminary analyses, we computed Pearson or Spearman correlations (depending on the distribution requirements) between dependent variables and age, duration of illness, PANSS, IDS-C30, and BDI-II. ANOVA (or Mann-Whitney *U* tests if there were deviations from normality assumption) was performed to test for associations between neuropsychological variables and sex, education, treatment setting, and medication (total number of antidepressants, antipsychotics, mood stabilizers, and tranquilizers). As in patients neurocognitive test results correlated higher with PANSS than with BDI-II and IDS-C_30_, we only used PANSS as covariate in ANCOVA intending to avoid multiple adjustment for these intervening variables which could falsify the results. For details between intervening variables and their correlations with neurocognitive tests see Supplementary Table 1. No systematic relationship was found between dependent variables and medication. If results of preliminary analyses were not significant, the variable was not included as covariate in further analyses.

As the main analytic method, ANCOVA was performed in two models: Model 1 was adjusted for the covariate age, and sex was added as a between-subject factor if it showed a significant effect in the preliminary analysis; model 2 used the same design, but education, treatment setting, and PANSS total score were included in patients and education was included in controls. The two-model-approach was performed to investigate the mediating factors education, treatment setting, and symptoms severity. In subsequent analyses, the delineated ANCOVA design was used separately for each of the five ELS types. Pearson or Spearman correlations were computed between the *total ELS load* score and the dependent variables.

As a post hoc sensitivity analysis, we calculated the effect size that could be detected for significance level *α* = 0.005 (0.05/10), adjusted for the number of dependent neuropsychological variables, with power 1 − *β* = 0.8. The power analysis was performed with G*Power 3.1.9 by procedure ANCOVA (Faul et al., 2007); fixed effects, main effects, and interactions; numerator degree of freedom = 1, number of groups = 2, and covariates = 1; and the achieved sample sizes of *n* = 197 for schizophrenia patients and *n* = 194 for controls. With these assumptions, the sensitivity analysis found that medium effect sizes of *f* = 0.263 (schizophrenia patients) and *f* = 0.265 (controls) could be assessed.

The significance level was generally set to *α* = 0.05. For analyses of neuropsychological variables, an adjusted significance level of *α* = 0.005 was assumed (Bonferroni correction). In the tables, numerical differences are highlighted also if *p* > 0.005 but *p* < 0.05, even though such results are nonsignificant. For the analyses of the five ELS items, a Bonferroni corrected significance level of *α* = 0.01 was applied.

Statistical analyses were performed with IBM SPSS statistics 25.

## 3 Results

### 3.1 Sociodemographic and clinical data

At baseline, the mean (SD) age of patients was 42.9 (12.0) years, and of controls, 38.5 (16.4) years; 66.0 % of patients and 38.6 % of controls were male (Table 1).

**Table 1:** Sociodemographic details, history of illness, and psychopathology of patients with schizophrenia (early life stress [ELS], *n* = 112; no ELS, *n* = 103) and healthy controls (ELS, *n* = 49; no ELS, *n* = 148).

	Patients Mean (SD) or n (%)				Controls Mean (SD) or n (%)			
	No ELS	ELS	Statistics	*p*	No ELS	ELS	Statistics	*p*
Age, m (SD), years	41.4 (11.8)	44.2 (12.1)	*F*(1, 213) = 2.92	0.089	36.7 (16.4)	43.3 (16.1)	*F*(1, 195) = 5.67	0.018⁎
Sex: male, n, (%)	72 (70.0)	70 (62.5)	*X*^2^(1) = 1.31	0.252	56 (37.8)	20 (40.8)	*X*^2^(1) = 0.14	0.710
Marital status: separated/divorced, n (%)	8 (7.8)	27 (24.1)	*X*^2^(2) = 11.02	0.004⁎	10 (6.8)	8 (16.3)	*X*^2^(2) = 4.04	0.133
Partnership: single, n (%)	65 (63.1)	73 (65.2)	*X*^2^(1) = 0.16	0.690	43 (29.0)	17 (34.7)	*X*^2^(1) = 0.55	0.457
No. of children, m (SD)	0.41 (0.78)	0.59 (1.07)	*F*(1, 209) = 1.94	0.165	0.53 (1.02)	0.60 (0.77)	*F*(1, 191) = 0.14	0.706
No. of sisters, m (SD)	0.67 (0.94)	0.81 (0.90)	*F*(1, 190) = 1.23	0.268	0.54 (0.70)	0.86 (1.14)	*F*(1, 192) = 5.20	0.024⁎
No. of brothers, m (SD)	0.86 (0.80)	0.92 (0.96)	*F*(1, 197) = 0.22	0.638	0.68 (0.88)	0.92 (1.08)	*F*(1, 191) = 2.37	0.126
Living alone, n (%)	48 (46.6)	57 (50.9)	*X*^2^(1) = 4.00	0.529	36 (24.3)	16 (32.6)	*X*^2^(1) = 1.31	0.252
Education: high school, n (%)	50 (48.5)	40 (35.7)	*X*^2^(2) = 4.82	0.090	130 (87.2)	32 (65.3)	*X*^2^(2) = 12.31	0.002⁎
Professional degree: academic, n (%)	18 (17.5)	11 (9.8)	*X*^2^(3) = 3.37	0.338	71 (47.6)	14 (28.6)	*X*^2^(3) = 13.72	0.003⁎
Currently without paid employment, n (%)	61 (59.2)	78 (69.6)	*X*^2^(1) = 2.30	0.130	45 (30.4)	11 (22.4)	*X*^2^(1) = 1.20	0.273
Absence from work in past 5 years, m (SD), mo	12.40 (16.37)	13.79 (17.38)	*F*(1, 98) = 0.17	0.681	0.03 (0.29)	0.24 (0.69)	*F*(1, 134) = 6.26	0.014⁎
Family member affected by psychiatric disorder: yes, n (%)	64 (62.1)	80 (71.4)	*X*^2^(1) = 2.91	0.008⁎	73 (49.3)	26 (53.1)	*X*^2^(1) = 0.17	0.680
Current treatment: inpatient/day patient, n (%)	35 (34.0)	50 (44.6)	*X*^2^(3) = 3.99	0.263	n.a.	n.a.		
Duration of illness, m (SD), years	12.5 (8.6)	15.8 (11.3)	*F*(1, 209) = 5.56	0.019⁎	n.a.	n.a.		
Lifetime alcohol dependency: yes, n, (%)	7 (6.8)	15 (13.4)	*X*^2^(1) = 2.48	0.115	0	0		
Use of illicit drugs: yes, n (%)	51 (49.5)	51 (45.5)	*X*^2^(1) = 0.04	0.837	75 (50.7)	22 (44.9)	*X*^2^(1) = 0.62	0.433
PANSS total sum score, m (SD)	52.73 (16.38)	58.38 (21.44)	*F*(1, 200) = 4.376	0.038⁎	30.59 (1.14)	30.84 (1.16)	*F*(1, 120) = 0.98	0.324
IDS-C_30_ sum score, m (SD)	22.21 (9.12)	13.33 (11.43)	*F*(1, 186) = 1.93	0.166	2.80 (2.73)	3.56 (3.67)	*F*(1, 171) = 1.42	0.236
BDI-II sum score, m (SD)	10.67 (9.19)	11.98 (11.35)	*F*(1, 186) = 0.76	0.385	2.15 (3.05)	3.76 (4.99)	*F*(1, 187) = 6.88	0.009⁎
CGI, m (SD)	3.97 (1.14)	4.32 (0.92)	*F*(1, 211) = 6.02	0.015⁎	n.a.	n.a.		
GAF, m (SD)	56.75 (13.30)	51.89 (14.09)	*F*(1, 211) = 6.67	0.001⁎	86.64 (5.75)	84.03 (5.54)	*F*(1, 129) = 4.70	0.032⁎
Suicidal ideation: yes, n (%)	73 (70.9)	83 (74.1)	*X*^2^(1) = 0.57	0.449	0	0		

BDI-II, Beck Depression Inventory II; CGI, Clinical Global Impression Scale; ELS, early life stress; GAF, Global Assessment of Functioning; IDS-C_30_, Clinician-rated Inventory of Depressive Symptomatology; m, mean; mo, months; n.a., not applicable; no ELS, participants reporting no significant early life stress; PANSS, Positive and Negative Syndrome Scale; SD, standard deviation.

⁎ *p* value < 0.05.

In patients, high ELS score was significantly correlated with more severe schizophrenia symptoms (*r* = 0.191, *df* = 200, *p* = 0.007), greater illness severity (*r* = 0.185, *df* = 208, *p* = 0.007), and lower level of functioning (*r* = −0.191, *df* = 208, *p* = 0.006). Healthy controls showed no significant associations between ELS score and psychopathology (depressive symptoms) or functioning (Table 2). In patients (F(2, 201) = 3.060, *p* = 0.049) as well as in controls (*F*(2, 191) = 9.56, *p* < 0.001), subjects with low education showed higher ELS scores than patients with high education.

**Table 2:** Correlations (Spearman's rho and Pearson) between early life stress score and cognitive performance and psychopathology/functioning in patients with schizophrenia and healthy controls.

			Patients			Controls		
			*n*	*r*	*p*	*n*	*r*	*p*
*Cognitive performance*								
Learning and memory	Verbal learning	VLMT correct words, sum 1 to 5 (words)	195	−0.153^a^	0.033#	194	−0.302^a^	<0.001⁎
	Consolidation	VLMT immediate loss of recalled words (words)	191	0.103^b^	0.156	193	0.041^b^	0.575
	Long-term memory	VLMT loss of recalled words after 25 min (words)	186	0.149^b^	0.043#	191	0.146^b^	0.023#
	Recognition	VLMT recognition (words)	185	−0.027^b^	0.720	190	−0.154^b^	0.033#
	Short-term memory	Digit Span forward (correct numbers)	202	−0.117^a^	0.098	196	−0.233^b^	0.001⁎
Executive function	Working memory	Digit Span backward (correct numbers)	202	−0.120^a^	0.090	195	−0.176^a^	0.014⁎⁎
	Task switching 1	TMT B (seconds)	198	0.181^a^	0.011#	195	0.273^a^	<0.001⁎
	Task switching 2	TMT B - TMT A (seconds)	198	0.090^a^	0.209	194	0.200^a^	0.005⁎
Psychomotor speed	Psychomotor speed 1	TMT A (seconds)	206	0.273^a^	<0.001⁎	196	0.221^a^	0.002⁎
	Psychomotor speed 2	Digit Symbol Test (symbols)	192	−0.174^a^	0.016⁎⁎	194	−0.233^a^	0.002⁎

*Psychopathology and functioning*								
Psychotic symptoms	PANSS sum score		202	0.191^a^	0.007⁎⁎	135	0.087^a^	0.314
Depressive symptoms	BDI-II sum score		192	0.134^b^	0.063	192	0.181^b^	0.012#
	IDS-C_30_ sum score		192	0.178^b^	0.014#	136	0.086^b^	0.322
Illness severity	CGI score		210	0.185^b^	0.007⁎⁎			
Functioning	GAF score		210	−0.191^a^	0.006⁎⁎	140	−0.149^a^	0.080

BDI-II, Beck Depression Inventory-II; CGI, Clinical Global Impression Scale; GAF, Global Assessment Functioning; IDS-C_30_, Clinician-rated Inventory of Depressive Symptomatology; PANSS, Positive and negative syndrome scale; TMT, Trail Making Test; VLMT, Verbal Learning Memory Test.

⁎ p < 0.005 significant (Bonferroni correction for multiple testing).

⁎⁎ *p* < 0.01 significant (Bonferroni correction for multiple testing).

# *p* < 0.05 numerical but not significant differences.

a Pearson's correlation.

b Spearman's rho correlation.

### 3.2 Prevalence of ELS

Significantly more patients than controls reported ELS (*n* = 112 [52.1 %] vs *n* = 49 [24.9 %], respectively; *X*^2^(1) = 31.99; *p* < 0.001). In both groups, emotional abuse was the most frequently reported ELS type (patients, *n* = 63 [29.0 %]; controls, *n* = 20 [10.2 %]; *X*^2^(1) = 22.96; *p* < 0.001) and physical neglect, the least (patients, *n* = 34 [15.4 %]; controls, *n* = 7 [3.5 %]; *X*^2^(1) = 16.61; *p* < 0.001). Physical neglect was reported by significantly more male than female controls (*n* = 6 [7.9 %] vs *n* = 1 [0.8 %], respectively; *X*^2^(1) = 6.69, *p* = 0.010), but this was not the case in patients (men, *n* = 17 [11.5 %]; women, *n* = 17 [23.0 %]), so the Breslow-Day test showed significant inhomogeneity of odds ratios (*X*^2^(1) = 11.54; *p* = 0.001). In contrast, sexual abuse was reported by a significantly higher percentage of women than men in controls (*n* = 17 [15.7 %] vs *n* = 2 [2.6 %], respectively; *X*^2^(1) = 7.20; *p* = 0.007) and patients (*n* = 19 [26.0 %] vs *n* = 21 [14.7 %], respectively; *X*^2^(1) = 4.02; *p* = 0.045), and Cochran's test showed significance for conditional independence (*X*^2^(1) = 10.33, *p* = 0.001).

### 3.3 Neurocognition

#### 3.3.1 CCS

##### 3.3.1.1 ELS in general

**Fig. 1:**
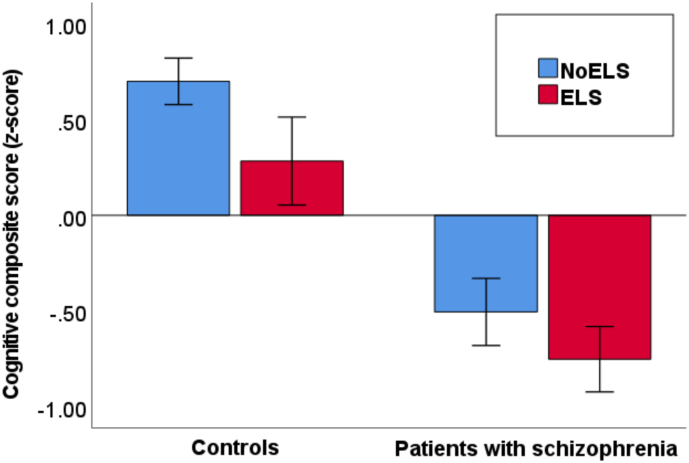
Comparison of cognitive composite score in patients with schizophrenia and controls with and without early life stress. Legend: Cognitive composite scores (z-scores) are shown for controls and patients with schizophrenia with and without early life stress (ELS). Error bars show 95 % confidence intervals.

In model 1, CCS was numerically lower in patients with (mean −0.75 [SD 0.82]) than in those without ELS (−0.50 [0.80]; *F*(1, 172) = 3.0; *p* = 0.085) and significantly lower in controls with (0.28 [0.77]) than in those without ELS (0.70 [0.73]; *F*(1, 183) = 5.7; *p* = 0.018; Fig. 1). CCS was much larger in controls (0.60 [0.76]) than in patients (−0.63 [0.82]; *F*(1, 356) = 185.5, *p* < 0.001). In model 2, the score was not significantly associated with ELS in patients (*F*(1, 155) = 0.4; *p* = 0.51) or controls (*F*(1, 177) = 1.2; *p* = 0.27).

##### 3.3.1.2 ELS subtypes

In model 1, CCS was significantly lower in patients with physical neglect (−1.36 [0.65]) than in those without it (−0.52 [0.79]; *F*(1, 171) = 14.7; *p* < 0.001), in controls with physical neglect (−0.23 [0.90]) than in those without it (0.63 [0.74]; *F*(1, 183) = 6.1; *p* = 0.015), and in controls with physical abuse (−0.24 [0.74]) than in those without it (0.69 [0.71]; *F*(1, 183) = 12.1; *p* = 0.001). However, none of these associations remained significant in model 2.

##### 3.3.1.3 ELS score

In both groups, CCS was significantly negatively correlated with a high ELS score (patients, *r* = −0.163, *df* = 168, *p* = 0.033; controls, *r* = −0.305, *df* = 184, *p* < 0.001). In patients, this score was not significantly correlated with ELS after adjustment for PANSS total score (*r* = −0.095, *df* = 159, *p* = 0.23) and age (*r* = −0.111, *df* = 165, *p* = 0.151), but in controls, it was still significantly correlated with ELS after adjustment for age (*r* = −0.200, *df* = 181, *p* = 0.006).

#### 3.3.2 Specific cognitive domains

##### 3.3.2.1 ELS in general

In model 1, controls with ELS showed significantly more impairment in learning and memory (*verbal learning*) than those without ELS (Table 3, *F*(1, 190) = 11.98, *p* = 0.001), but this result was not significant in model 2 (*F*(1, 184) = 3.98, *p* = 0.048). No other significant differences were found between the ELS and no-ELS groups.

**Table 3:** Results of analyses of covariance (model 1 and model 2) comparing cognitive performance between early life stress subtypes and no early life stress in patients and controls.

		Model 1					Model 2				
		No ELS Mean (SD)	ELS Mean (SD)	Statistics	Adjusted *r*2	*p*	No ELS Mean (SD)	ELS Mean (SD)	Statistics	Adjusted *r*2	*p*
*Patients*											
Learning and memory	Verbal learning	44.89 (10.76)	42.74 (11.81)	*F*(1, 197) = 0.81	0.088	0.369	45.01 (10.70)	42.69 (11.86)	*F*(1, 178) = 1.02	0.288	0.315
	Consolidation	2.31 (2.22)	2.44 (2.29)	*F*(1, 193) = 0.07	0.003	0.793	2.31 (2.25)	2.52 (2.26)	*F*(1, 175) = 0.15	0.010	0.702
	Long-term memory	2.71 (2.29)	3.01 (2.43)	*F*(1, 188) = 0.50	0.007	0.479	2.67 (2.32)	3.05 (2.42)	*F*(1, 171) = 1.31	0.016	0.254
	Recognition	10.51 (4.23)	10.66 (3.69)	*F*(1, 187) = 0.32	0.031	0.570	10.43 (4.25)	10.63 (3.73)	*F*(1, 169) = 2.63	0.087	0.106
	Short-term memory	9.36 (2.10)	8.85 (2.11)	*F*(1, 200) = 2.13	0.026	0.146	9.34 (2.05)	8.86 (2.09)	*F*(1, 184) = 1.21	0.106	0.272
Executive function	Working memory	6.12 (2.20)	5.64 (2.17)	*F*(1, 204) = 1.91	0.010	1.169	6.20 (2.21)	5.67 (2.15)	*F*(1, 184) = 0.27	0.157	0.605
	Task switching 1	80.60 (45.62)	92.83 (45.81)	*F*(1, 200) = 4.81	0.142	0.029#	78.77 (41.09)	92.87 (46.35)	*F*(1, 181) = 3.26	0.345	0.073
	Task switching 2	48.77 (38.38)	54.37 (40.11)	*F*(1, 200) = 0.67	0.073	0.413	46.85 (32.70)	54.19 (40.72)	*F*(1, 181) = 0.28	0.256	0.595
Psychomotor speed	Psychomotor speed 1	32.86 (15.45)	39.02 (18.19)	*F*(1, 208) = 5.50	0.176	0.020⁎	33.03 (15.84)	39.40 (38.34)	*F*(1, 188) = 2.74	0.306	0.099
	Psychomotor speed 2	58.88 (18.74)	52.81 (18.30)	*F*(1, 192) = 6.70	0.073	0.010#	57.91 (18.99)	52.91 (18.54)	*F*(1, 172) = 1.10	0.190	0.296

*Controls*											
Learning and memory	Verbal learning	60.51 (8.52)	54.85 (10.14)	*F*(1, 190) = 11.98	0.362	0.001⁎	60.47 (8.53)	54.62 (10.12)	*F*(1, 184) = 3.98	0.407	0.048#
	Consolidation	1.03 (1.46)	1.33 (2.50)	*F*(1, 191) = 0.31	0.028	0.577	1.03 (1.46)	1.38 (2.51)	*F*(1, 185) = 2.61	0.090	0.108
	Long-term memory	1.16 (2.26)	1.71 (2.52)	*F*(1, 189) = 1.84	0.004	0.177	1.13 (2.23)	1.77 (2.51)	*F*(1, 183) = 4.95	0.018	0.027#
	Recognition	13.27 (3.14)	13.04 (2.53)	*F*(1, 188) = 0.10	0.082	0.749	13.26 (3.14)	13.02 (2.55)	*F*(1, 182) = 0.41	0.084	0.521
	Short-term memory	11.08 (2.19)	10.20 (2.38)	*F*(1, 194) = 2.66	0.135	0.105	11.07 (2.20)	10.21 (2.41)	*F*(1, 188) = 1.11	0.186	0.294
Executive function	Working memory	8.23 (2.54)	7.31 (2.20)	*F*(1, 193) = 2.90	0.098	0.090	8.24 (2.55)	7.34 (2.22)	*F*(1, 187) = 0.95	0.106	0.332
	Task switching 1	51.90 (24.36)	60.24 (27.77)	*F*(1, 191) = 0.86	0.361	0.355	51.97 (24.43)	60.23 (28.06)	*F*(1, 185) = 0.40	0.403	0.526
	Task switching 2	28.56 (18.77)	33.67 (22.99)	*F*(1, 189) = 0.02	0.227	0.902	28.57 (18.83)	33.43 (23.17)	*F*(1, 183) = 0.03	0.288	0.862
Psychomotor speed	Psychomotor speed 1	23.39 (10.78)	26.57 (9.95)	*F*(1, 193) = 1.66	0.285	0.199	23.45 (10.79)	26.79 (9.93)	*F*(1, 194) = 1.03	0.296	0.312
	Psychomotor speed 2	88.12 (18.14)	79.18 (16.98)	*F*(1, 190) = 5.45	0.447	0.021#	88.16 (18.19)	79.06 (17.14)	*F*(1, 184) = 0.94	0.463	0.334

Model 1: adjusted for age and sex (if necessary).

Model 2: adjusted for age sex (if necessary), educational level, treatment setting, and Positive and Negative Syndrome Scale.

Verbal learning, sum of correct words in rounds 1 to 5 of VLMT (words); consolidation, immediate loss of recalled words in VLMT (words); long-term memory, loss of recalled words after 25 min in VLMT (words); recognition, recognition of words in VLMT (words); short-term memory, Digit Span forward (correct numbers); working memory, Digit Span backward (correct numbers); task switching 1, TMT B (seconds); task switching 2, TMT B - TMT A (seconds); psychomotor speed 1, TMT A (seconds); psychomotor speed 2, Digit Symbol Test (symbols).

ELS, participants with early life stress; no ELS, participants reporting no early life stress; SD, standard deviation; TMT, Trail Making Test; VLMT, Verbal Learning and Memory Test.

⁎ *p* < 0.005 significant (Bonferroni correction for multiple testing).

# *p* < 0.05 numerical but not significant differences.

##### 3.3.2.2 ELS subtypes

As shown in the Supplementary Table 2, in model 1 patients with sexual abuse showed significantly worse performance in one item of the domain *psychomotor speed* (*F*(1, 196) = 8.79, *p* = 0.003) than patients without sexual abuse and patients with physical neglect showed greater impairment than patients without it in *verbal learning* (*F*(1, 196) = 11.45, p = 0.001)*, short term memory F*(1, 196) = 11.45, *p* = 0.001 and *working memory* (*F*(1, 203) = 8.79, *p* = 0.003). In controls, emotional neglect was significantly associated with more cognitive deficits in the domain *consolidation* (*F*(1, 191) = 8.49, *p* = 0.004) and physical abuse was significantly associated with more cognitive deficits in the domain *verbal learning* (*F*(1, 190) = 8.51, p = 0.004) comparing patients with and without the respective ELS subtype.

In model 2, no statistically significant association between any ELS subtype and cognitive impairment was seen in patients. In controls, emotional neglect, physical abuse, and emotional abuse were significantly associated with *consolidation* (all *F* ≥ 12.56, *p* ≤ 0.001) and *long-term memory* (all *F* ≥ 8.76, *p* ≤ 0.003) when comparing those with and without the respective ELS subtype; however, sexual abuse and physical neglect showed no significant associations with cognitive performance.

##### 3.3.2.3 ELS score

In patients, higher ELS score was significantly correlated with greater impairment in *psychomotor speed* (Table 2, *r* = 0.273, *df* = 204, *p* < 0.001), and in controls, with greater deficits in *verbal learning* (*r* = −0.302, *df* = 192, *p* < 0.001), *short-term memory* (*r* = −0.233, *df* = 194, *p* = 0.001), *psychomotor speed* (all |*r*| ≥ 0.221, *df* ≥ 192, *p* ≤ 0.002), and *executive functioning* (TMT-B: *r* = 0.273, *df* = 193, *p* < 0.001).

## 4 Discussion

This study investigated whether ELS is associated with neurocognitive performance in patients with schizophrenia and healthy controls. Independent of ELS, patients had lower cognitive performance on neuropsychological tests, which is in line with previous research: Cognitive deficits are a robust phenotype in schizophrenia, and patients have a 1.5- to 2.5-point standard deviation CCS deficit compared with healthy controls (Bilder et al., 2000; Keefe, 2014).

Our main finding was the significant association of ELS and cognitive deficits (CCS) in controls but not in patients. The finding that ELS was significantly associated with impaired general neurocognitive performance in controls is consistent with existing literature (Mills et al., 2011; Perez and Widom, 1994; Sideli et al., 2014; van Os et al., 2017). Research results showed a more variable association between ELS and cognitive performance in patients with schizophrenia than in healthy controls (Dauvermann and Donohoe, 2018). In patients, we hypothesize that disease-related cognitive deficits may mask the effect of ELS on cognitive impairment: Deficits are heterogenous and can occur in late childhood or early adolescence, before the onset of schizophrenia (Fuller et al., 2002; Sideli et al., 2014), and this premorbid impairment may attenuate the effect of ELS on cognitive function. And, it emphasizes the theory that ELS has a profound detrimental effect on brain development (Popovic et al., 2019) Furthermore, cholinergic effects of antipsychotics could have weakened the impact in patients (Minzenberg et al., 2004).

To investigate whether education and symptom burden mediate the effect of ELS on cognitive performance, we applied the two-model-approach. In model 1, in both groups the CCS was lower in those with than in those without ELS, but this association was not found in model 2 (after adjustment for education, treatment setting, PANSS). This difference suggests that other factors, such as education and symptom burden, interfere with cognitive performance; it is in line with Aas et al. indicating that effects of ELS on cognitive performance are mediated by lower general intelligence and educational attainment (Aas et al., 2012). Lack of social integration due to limited participation in education and career development may also contribute to this observation. Moreover, patients with a history of ELS have more symptoms than those without ELS (Carbone et al., 2019; Garcia et al., 2016; Trotta et al., 2015), so the direction of the relationship remains unclear. Cognitive deficits and psychotic symptoms share genetic and environmental aspects of etiology (Reichenberg et al., 2019).

In our ELS subtype analyses, physical neglect and abuse were closely associated with impaired neurocognitive performance. Kilian et al. showed that physical neglect is a significant predictor of impaired social cognition in patients with schizophrenia and controls (Kilian et al., 2018), and Mørkved et al. showed that it is associated with worse attention and working memory in patients (Mørkved et al., 2020). Of interest is that physical neglect is often underestimated and overlooked in clinical assessments (Larsson et al., 2013). Consistent with existing literature, our results showed that ELS is more common in patients with schizophrenia than in healthy controls (Bonoldi et al., 2013; Larsson et al., 2013) and that different ELS subtypes frequently co-occur (Kessler et al., 2010). The high prevalence of ELS in our control group is concerning and relevant for prevention programs against childhood trauma (Saunders and Adams, 2014). Education as mediating factor for cognitive deficits could raise the need for targeted school support. It is important to note that despite notable trauma and cognitive deficits, healthy controls did not present to professional care and were not identified as clinically ill in the study screening. Awareness, prevention, and early intervention programs are required to mitigate the long-term consequences of ELS.

The sub-analyses of cognitive domains showed that ELS affects different domains in patients and controls, but no clear pattern emerged. Previous research revealed similar findings (Dauvermann and Donohoe, 2018). These inconsistencies could be due to individual stress responses, different coping strategies, and variability of cognitive performance in schizophrenia, which is partly explained by genetic risk (Comes et al., 2019; Engen et al., 2020; Popovic et al., 2019; Richards et al., 2019; Schaupp et al., 2018). In addition, protective and unfavorable environmental factors and related epigenetic effects might also play a role (Brown, 2011; Popovic et al., 2019). In our study, the small number of cases in the sub-analyses might also have contributed to our inconsistent findings.

### 4.1 Limitations

Our study has some limitations. ELS data were obtained retrospectively and prone to recall bias. Moreover, the CTS does not provide detailed information on the frequency, severity, age of trauma occurrence, or traumatic experience in adulthood. Age and sex were not well balanced, our patients mainly had a long illness duration, which might have meant more advanced cognitive decline. Patients with first-episode schizophrenia were underrepresented, and the variability in cognitive data was rather small. Only the total number of prescribed medications was included, detailed data, e.g. chlorpromazine equivalents, were lacking. Information on traumatic brain injury or cognitive impairment due to other neurological illnesses is not considered. Our neurocognitive assessment covers several cognitive domains, but could be even more comprehensive, e.g. nonverbal, visual measures and premorbid intellectual functioning. Although we have a solid sample size, the power could have been better. Ideally, analyses should be enriched by multi-method approaches, including imaging, biomarker research, epigenetics, and genetics.

## 5 Conclusion

ELS is more strongly associated with cognitive deficits in healthy controls than in patients and is associated with worse cognitive performance and social functioning in both. Patients with ELS have more pronounced schizophrenia symptoms. Our results are clinically relevant because they indicate that regular, standardized assessment of cognitive deficits and recording of ELS are important in patients with schizophrenia. Patients with schizophrenia and ELS should receive trauma-focused therapies (Coughlan and Cannon, 2017), as well as special cognitive training regardless of ELS experiences (Prikken et al., 2019). The results in the healthy control group should lead to a highlighted attention to the relationship between ELS and cognition. Early trauma prevention could lead to minimizing cognitive deficits even in subclinically affected individuals or people at ultra-high risk for psychosis.

## Ethical standards

Approval by Ethics Committee, Medical Faculty, LMU Munich, project number: 17-13. The study has been performed in accordance with the ethical standards laid down in the 1964 Declaration of Helsinki and its later amendments. Written informed consent was obtained from the participants of the study.

## CRediT authorship contribution statement

**Fanny Senner:** Conceptualization, Investigation, Formal analysis, Writing – original draft, Methodology, Visualization. **Thomas Schneider-Axmann:** Writing – review & editing, Formal analysis, Methodology, Visualization. **Lalit Kaurani:** Writing – review & editing. **Jörg Zimmermann:** Resources. **Jens Wiltfang:** Resources. **Martin von Hagen:** Resources. **Thomas Vogl:** Investigation, Writing – review & editing. **Carsten Spitzer:** Resources. **Simon Senner:** Writing – review & editing. **Eva C. Schulte:** Investigation, Data curation, Writing – review & editing. **Max Schmauß:** Resources. **Sabrina K. Schaupp:** Investigation, Writing – review & editing. **Jens Reimer:** Resources. **Daniela Reich-Erkelenz:** Writing – review & editing. **Sergi Papiol:** Data curation, Writing – review & editing. **Mojtaba Oraki Kohshour:** Writing – review & editing. **Fabian U. Lang:** Resources. **Carsten Konrad:** Resources. **Sophie-Kathrin Kirchner:** Writing – review & editing. **Janos L. Kalman:** Investigation, Data curation, Writing – review & editing. **Georg Juckel:** Resources. **Maria Heilbronner:** Investigation. **Urs Heilbronner:** Investigation, Data curation, Writing – review & editing. **Christian Figge:** Resources. **Ruth E. Eyl:** Writing – review & editing. **Detlef Dietrich:** Resources. **Monika Budde:** Investigation, Data curation, Writing – review & editing. **Ion-George Angelescu:** Resources. **Kristina Adorjan:** Investigation, Data curation, Writing – review & editing. **Andrea Schmitt:** Supervision, Writing – review & editing. **Andre Fischer:** Writing – review & editing. **Peter Falkai:** Conceptualization, Funding acquisition, Project administration. **Thomas G. Schulze:** Conceptualization, Funding acquisition, Project administration, Writing – review & editing.

## Conflict of interest

Ion-George Anghelescu has been member of advisory boards and received speakers honoraria of Janssen-Cilag and Dr. Willmar Schwabe and received speakers honoraria of Recordati. P. Falkai has been an honorary speaker for AstraZeneca, Bristol Myers Squibb, Lilly, Essex, GE Healthcare, GlaxoSmithKline, Janssen Cilag, Lundbeck, Otsuka, Pfizer, Servier, and Takeda and has been a member of the advisory boards of Janssen-Cilag, AstraZeneca, Lilly, Lundbeck, Richter, Recordati and Boehringer Ingelheim. C. Konrad received fees for an educational program from Aristo Pharma, Janssen-Cilag, Lilly, MagVenture, Servier, and Trommsdorff as well as travel support and speakers honoraria from Aristo Pharma, Janssen-Cilag, Lundbeck, Neuraxpharm and Servier. A. Schmitt was an honorary speaker for TAD Pharma and Roche and a member of Roche advisory boards. J. Wiltfang has been an honorary speaker for Actelion, Amgen, Beeijing Yibai Science and Technology Ltd., Janssen Cilag, Med Update GmbH, Pfizer, Roche Pharma, and has been a member of the advisory boards of Abbott, Biogen, Boehringer Ingelheim, Lilly, MSD Sharp & Dohme, and Roche Pharma and receives fees as a consultant for Immungenetics and Roboscreen. All other authors report no conflicts of interest.

## Appendix A Supplementary data

Supplementary tables Image 1

Supplementary material Image 2
