# Supplementary material for: Association of early life stress and cognitive performance in patients with schizophrenia and healthy controls
Source: Schizophr Res Cogn. 2023 Feb 11;32:100280. doi: 10.1016/j.scog.2023.100280 (PMC9945796; doi:10.1016/j.scog.2023.100280)
Supplement: Supplementary file 1 — Supplementary tables [file mmc1.docx]

**Supplementary Table 1.**

Correlations between intervening variables and correlations between neurocognitive testing in patients with and without ELS

|  | **Patients: no ELS** | | | | |  |  |  |  | **Patients: ELS** | | |  |  |  |
| --- | --- | --- | --- | --- | --- | --- | --- | --- | --- | --- | --- | --- | --- | --- | --- |
|  |  |  | |  | |  |  |  |  |  |  |  |  |  |  |
|  |  | age (years) | |  | |  |  |  |  |  | age (years) |  |  |  |  |
| duration of illness (years) | *r* | 0.529^**^ | |  | |  |  |  |  | *r* | 0.661^**^ |  |  |  |  |
|  | *p* | <0.001 | |  | |  |  |  |  | *p* | <0.001 |  |  |  |  |
|  | *N* | 102 | |  | |  |  |  |  | *N* | 109 |  |  |  |  |
|  |  |  | |  | |  |  |  |  |  |  |  |  |  |  |
|  |  | PANSS total sum | | IDS-C_30_ | |  |  |  |  |  | PANSS total sum | IDS-C_30_ |  |  |  |
| IDS-C_30_ sum | *r* | 0.549^**^ | |  | |  |  |  |  | *r* | 0.497^**^ |  |  |  |  |
|  | *p* | <0.001 | |  | |  |  |  |  | *p* | <0.001 |  |  |  |  |
|  | *N* | 88 | |  | |  |  |  |  | *N* | 102 |  |  |  |  |
| BDI-II sum | *r* | 0.433^**^ | | 0.716^**^ | |  |  |  |  | *r* | 0.543^**^ | 0.743^**^ |  |  |  |
|  | *p* | <0.001 | | <0.001 | |  |  |  |  | *p* | <0.001 | <0.001 |  |  |  |
|  | *N* | 91 | | 85 | |  |  |  |  | *N* | 97 | 95 |  |  |  |
|  |  |  | |  | |  |  |  |  |  |  |  |  |  |  |
|  | **Patients: no ELS** | | | | |  |  |  |  | **Patients: ELS** | | |  |  |  |
|  |  | age (years) | duration of illness (years) | | PANSS total sum | | IDS-C_30_ | BDI-II sum |  |  | age (years) | duration of illness (years) | PANSS total sum | IDS-C_30_ | BDI-II sum |
| Verbal learning | *r* | -0.358^**^ | -0.208^*^ | | -0.168 | | -0.047 | -0.292^**^ |  | *r* | -0.252^*^ | -0.104 | -0.396^**^ | -0.339^**^ | -0.246^*^ |
|  | *p* | <0.001 | 0.041 | | 0.107 | | 0.668 | 0.005 |  | *p* | 0.010 | 0.295 | <0.001 | 0.001 | 0.017 |
|  | *N* | 97 | 97 | | 93 | | 85 | 93 |  | *N* | 103 | 103 | 99 | 96 | 94 |
| Consolidation | *r* | 0.186 | 0.059 | | 0.052 | | 0.009 | 0.075 |  | *r* | 0.097 | -0.033 | 0.001 | 0.001 | 0.037 |
|  | *p* | 0.069 | 0.568 | | 0.623 | | 0.935 | 0.476 |  | *p* | 0.338 | 0.744 | 0.995 | 0.991 | 0.724 |
|  | *N* | 96 | 96 | | 92 | | 84 | 92 |  | *N* | 100 | 100 | 97 | 93 | 92 |
| Long-term memory | *r* | 0.153 | 0.013 | | 0.056 | | -0.037 | 0.022 |  | *r* | 0.212 | 0.067 | 0.077 | 0.041 | 0.100 |
|  | *p* | 0.141 | 0.898 | | 0.597 | | 0.741 | 0.841 |  | *p* | 0.037 | 0.512 | 0.456 | 0.700 | 0.351 |
|  | *N* | 94 | 94 | | 90 | | 82 | 90 |  | *N* | 97 | 97 | 95 | 90 | 89 |
| Recognition | *r* | -0.157 | 0.015 | | -0.101 | | -0.082 | -0.187 |  | *r* | -0.261 | -0.166 | -0.259 | -0.061 | -0.062 |
|  | *p* | 0.134 | 0.886 | | 0.351 | | 0.468 | 0.081 |  | *p* | 0.009 | 0.103 | 0.011 | 0.566 | 0.559 |
|  | *N* | 92 | 92 | | 88 | | 81 | 88 |  | *N* | 98 | 98 | 95 | 91 | 90 |
| Short-term memory | *r* | -0.145 | -0.175 | | -0.100 | | 0.014 | 0.014 |  | *r* | -0.146 | <0.001 | -0.247 | -0.161 | -0.160 |
|  | *p* | 0.150 | 0.082 | | 0.334 | | 0.893 | 0.894 |  | *p* | 0.132 | 0.996 | 0.012 | 0.109 | 0.119 |
|  | *N* | 100 | 100 | | 96 | | 88 | 94 |  | *N* | 107 | 107 | 103 | 100 | 96 |
| Working memory | *r* | -0.119 | -0.031 | | -0.184 | | 0.071 | 0.019 |  | *r* | -0.104 | <0.001 | -0.199 | -0.154 | -0.045 |
|  | *p* | 0.239 | 0.762 | | 0.073 | | 0.510 | 0.855 |  | *p* | 0.286 | 0.999 | 0.044 | 0.127 | 0.664 |
|  | *N* | 100 | 100 | | 96 | | 88 | 94 |  | *N* | 107 | 107 | 103 | 100 | 96 |
| Task switching 1 | *r* | 0.380^**^ | 0.229^*^ | | 0.204^*^ | | -0.017 | 0.130 |  | *r* | 0.322^**^ | 0.205^*^ | 0.279^**^ | 0.235^*^ | 0.059 |
|  | *p* | <0.001 | 0.023 | | 0.048 | | 0.879 | 0.218 |  | *p* | 0.001 | 0.037 | 0.005 | 0.020 | 0.572 |
|  | *N* | 99 | 99 | | 95 | | 87 | 92 |  | *N* | 104 | 104 | 100 | 97 | 95 |
| Task switching 2 | *r* | 0.298^**^ | 0.143 | | 0.164 | | -0.013 | 0.144 |  | *r* | 0.254^**^ | 0.197^*^ | 0.144 | 0.121 | -0.092 |
|  | *p* | 0.003 | 0.157 | | 0.112 | | 0.905 | 0.170 |  | *p* | 0.009 | 0.045 | 0.154 | 0.238 | 0.376 |
|  | *N* | 99 | 99 | | 95 | | 87 | 92 |  | *N* | 104 | 104 | 100 | 97 | 95 |
| Psychomotor speed 1 | *r* | 0.471^**^ | 0.278^**^ | | 0.330^**^ | | 0.055 | -0.001 |  | *r* | 0.325^**^ | 0.230^*^ | 0.365^**^ | 0.216^*^ | 0.228^*^ |
|  | *p* | <0.001 | 0.005 | | 0.001 | | 0.608 | 0.993 |  | *p* | 0.001 | 0.016 | <0.001 | 0.029 | 0.024 |
|  | *N* | 102 | 102 | | 98 | | 90 | 94 |  | *N* | 109 | 109 | 105 | 102 | 98 |
| Psychomotor speed 2 | *r* | -0.225^*^ | -0.099 | | -0.181 | | -0.242^*^ | -0.213^*^ |  | *r* | -0.182 | -0.178 | -0.298^**^ | -0.347^**^ | -0.182 |
|  | *p* | 0.029 | 0.345 | | 0.088 | | 0.029 | 0.047 |  | *p* | 0.066 | 0.073 | 0.003 | 0.001 | 0.082 |
|  | *N* | 94 | 94 | | 90 | | 82 | 87 |  | *N* | 103 | 103 | 99 | 96 | 92 |

** Correlation is significant at the 0.05 level (2-tailed)*

*** Correlation is significant at the 0.01 level (2-tailed)*

**^†^***Verbal learning, sum of correct words in rounds 1 to 5 of VLMT (words); consolidation, immediate loss of recalled words in VLMT (words); long-term memory, loss of recalled words after 25 minutes in VLMT (words); recognition, recognition of words in VLMT (words); short-term memory, Digit Span forward (correct numbers); working memory, Digit Span backward (correct numbers); task switching 1, TMT B (seconds); task switching 2, TMT B - TMT A (seconds); psychomotor speed 1, TMT A (seconds); psychomotor speed 2, Digit Symbol Test (symbols)*

*ELS, participants with early life stress; no ELS, participants reporting no significant early life stress*

**Supplementary Table 2.**

*Results of analyses of covariance (model 1 and model 2) comparing cognitive performance between early life stress subtypes and no early life stress in patients with schizophrenia and healthy controls*

|  |  |  |  | **Model 1** |  | **Model 2** |  |
| --- | --- | --- | --- | --- | --- | --- | --- |
| **ELS subtypes and types of cognitive performance** | **Domains of cognitive performance^†^** | No ELS  Mean (SD) | ELS  Mean (SD) | Statistics | *p* | Statistics | *p* |
|  |  | **Patients** | | | | | |
| **Emotional neglect** |  |  |  |  |  |  |  |
| Learning and memory | Verbal learning | 42.88 (11.50) | 43.42 (10.85) | *F*(1, 197) = 0.28 | 0.599 | *F*(1, 178) = 1.88 | 0.172 |
|  | Consolidation | 2.32 (2.32) | 2.59 (2.00) | *F*(1, 193) = 0.22 | 0.642 | *F*(1, 175) = 0.06 | 0.814 |
|  | Long-term memory | 2.75 (2.48) | 3.28 (1.80) | *F*(1, 188) = 0.97 | 0.325 | *F*(1, 171) = 0.37 | 0.370 |
|  | Recognition | 10.60 (4.04) | 10.55 (3.64) | *F*(1, 187) = 0.15 | 0.696 | *F*(1, 169) = 0.88 | 0.349 |
|  | Short-term memory | 9.10 (2.22) | 9.07 (1.74) | *F*(1, 204) = 0.09 | 0.770 | *F*(1, 184) = 0.00 | 0.950 |
| Executive function | Working memory | 5.98 (2.2.4) | 5.51 (2.01) | *F*(1, 204) = 1.04 | 0.308 | *F*(1, 184) = 1.13 | 0.290 |
|  | Task switching 1 | 85.60 (47.64) | 91.56 (39.56) | *F*(1, 200) = 0.55 | 0.458 | *F*(1, 181) = 0.42 | 0.520 |
|  | Task switching 2 | 51.32 (41.17) | 52.81 (31.67) | *F*(1, 200) = 0.08 | 0.784 | *F*(1, 181) = 0.13 | 0.720 |
| Psychomotor speed | Psychomotor speed 1 | 35.01 (16.50) | 39.66 (19.04) | *F*(1, 208) = 0.89 | 0.346 | *F*(1, 188) = 0.08 | 0.781 |
|  | Psychomotor speed 2 | 56.09 (18.96) | 54.42 (18.02) | *F*(1, 192) = 0.00 | 0.941 | *F*(1, 172) = 1.01 | 0.315 |
| **Physical abuse** |  |  |  |  |  |  |  |
| Learning and memory | Verbal learning | 43.95 (11.04) | 43.02 (12.64) | *F*(1, 195) = 0.02 | 0.894 | *F*(1, 176) = 0.70 | 0.403 |
|  | Consolidation | 2.33 (2.20) | 2.57 (2.48) | *F*(1, 191) = 0.20 | 0.656 | *F*(1, 173) = 0.13 | 0.908 |
|  | Long-term memory | 2.75 (2.26) | 3.27 (2.72) | *F*(1, 186) = 1.09 | 0.298 | *F*(1, 169) = 0.66 | 0.417 |
|  | Recognition | 10.47 (4.01) | 10.83 (3.77) | *F*(1, 185) = 0.84 | 0.362 | *F*(1, 167) = 2.67 | 0.104 |
|  | Short-term memory | 9.18 (2.18) | 8.79 (1.88) | *F*(1, 202) = 0.57 | 0.451 | *F*(1, 182) = 0.17 | 0.676 |
| Executive function | Working memory | 5.95 (2.33) | 5.60 (1.65) | *F*(1, 202) = 0.51 | 0.474 | *F*(1, 182) = 0.02 | 0.884 |
|  | Task switching 1 | 83.72 (44.95) | 99.44 (49.45) | *F*(1, 198) = 3.54 | 0.061 | *F*(1, 179) = 3.82 | 0.052 |
|  | Task switching 2 | 49.71 (38.55) | 59.54 (42.41) | *F*(1, 198) = 1.66 | 0.198 | *F*(1, 179) = 1.96 | 0.163 |
| Psychomotor speed | Psychomotor speed 1 | 34.48 (16.06) | 41.84 (20.17) | *F*(1, 206) = 3.99 | 0.047** | *F*(1, 186) = 3.08 | 0.081 |
|  | Psychomotor speed 2 | 55.94 (18.82) | 55.05 (18.95) | *F*(1, 190) = 0.02 | 0.877 | *F*(1, 170) = 0.04 | 0.836 |
| **Emotional abuse** |  |  |  |  |  |  |  |
| Learning and memory | Verbal learning | 43.63 (11.19) | 44.02 (11.95) | *F*(1, 195) = 0.16 | 0.680 | *F*(1, 176) = 0.49 | 0.483 |
|  | Consolidation | 2.42 (2.12) | 2.27 (2.59) | *F*(1, 191) = 0.21 | 0.644 | *F*(1, 173) = 0.29 | 0.594 |
|  | Long-term memory | 2.86 (2.23) | 2.96 (2.63) | *F*(1, 186) = 0.81 | 0.814 | *F*(1, 169) = 0.00 | 0.986 |
|  | Recognition | 10.40 (4.07) | 11.04 (3.63) | *F*(1, 185) = 1.18 | 0.279 | *F*(1, 167) = 1.17 | 0.280 |
|  | Short-term memory | 9.12 (2.22) | 9.10 (1.85) | *F*(1, 202) = 0.01 | 0.933 | *F*(1, 182) = 0.02 | 0.941 |
| Executive function | Working memory | 5.94 (2.19) | 5.75 (2.26) | *F*(1, 202) = 0.24 | 0.628 | *F*(1, 182) = 0.27 | 0.606 |
|  | Task switching 1 | 87.70 (51.26) | 85.36 (29.69) | *F*(1, 198) = 0.27 | 0.603 | *F*(1, 179) = 0.80 | 0.798 |
|  | Task switching 2 | 53.14 (43.70) | 48.50 (25.16) | *F*(1, 198) = 0.06 | 0.813 | *F*(1, 179) = 0.05 | 0.818 |
| Psychomotor speed | Psychomotor speed 1 | 35.22 (17.16) | 37.98 (17.41) | *F*(1, 206) = 1.20 | 0.275 | *F*(1, 186) = 0.65 | 0.652 |
|  | Psychomotor speed 2 | 55.98 (19.54) | 54.74 (16.98) | *F*(1, 190) = 0.04 | 0.846 | *F*(1, 170) = 0.29 | 0.592 |
| **Sexual abuse** |  |  |  |  |  |  |  |
| Learning and memory | Verbal learning | 44.71 (10.69) | 39.95 (13.32) | *F*(1, 196) = 0.16 | 0.157 | *F*(1, 177) = 0.01 | 0.934 |
|  | Consolidation | 2.43 (2.22) | 2.08 (2.36) | *F*(1, 192) = 1.28 | 0.258 | *F*(1, 174) = 1.86 | 0.175 |
|  | Long-term memory | 2.88 (2.33) | 2.72 (2.48) | *F*(1, 187) = 0.51 | 0.474 | *F*(1, 170) = 0.63 | 0.428 |
|  | Recognition | 10.54 (4.09) | 11.03 (3.08) | *F*(1, 186) = 1.69 | 0.195 | *F*(1, 168) = 4.62 | 0.033** |
|  | Short-term memory | 9.14 (2.06) | 8.82 (2.34) | *F*(1, 203) = 0.09 | 0.765 | *F*(1, 183) = 0.49 | 0.485 |
| Executive function | Working memory | 5.88 (2.13) | 5.71 (2.30) | *F*(1, 203) = 0.00 | 0.994 | *F*(1, 183) = 0.29 | 0.592 |
|  | Task switching 1 | 85.38 (47.31) | 91.58 (40.44) | *F*(1, 199) = 0.00 | 0.971 | *F*(1, 180) = 0.17 | 0.684 |
|  | Task switching 2 | 52.19 (40.81) | 50.03 (31.88) | *F*(1, 199) = 1.17 | 0.280 | *F*(1, 180) = 0.24 | 0.202 |
| Psychomotor speed | Psychomotor speed 1 | 34.38 (16.10) | 42.85 (19.95) | *F*(1, 207) = 3.57 | 0.060 | *F*(1, 187) = 1.17 | 0.281 |
|  | Psychomotor speed 2 | 57.84 (18.77) | 47.08 (16.22) | *F*(1, 191) = 8.79 | 0.003* | *F*(1, 171) = 3.67 | 0.057 |
| **Physical neglect** |  |  |  |  |  |  |  |
| Learning and memory | Verbal learning | 45.31 (11.00) | 35.88 (9.98) | *F*(1, 196) = 11.45 | 0.001* | *F*(1, 177) = 1.33 | 0.250 |
|  | Consolidation | 2.28 (2.28) | 2.93 (2.05) | *F*(1, 192) = 1.30 | 0.256 | *F*(1, 174) = 0.45 | 0.502 |
|  | Long-term memory | 2.67 (2.34) | 4.00 (2.24) | *F*(1, 187) = 5.65 | 0.018** | *F*(1, 170) = 3.67 | 0.057 |
|  | Recognition | 10.91 (3.85) | 8.64 (4.04) | *F*(1, 186) = 4.40 | 0.037** | *F*(1, 168) = 0.38 | 0.536 |
|  | Short-term memory | 9.31 (2.11) | 7.97 (1.85) | *F*(1, 203) = 8.21 | 0.005* | *F*(1, 183) = 3.46 | 0.065 |
| Executive function | Working memory | 5.09 (2.22) | 4.76 (1.74) | *F*(1, 203) = 8.79 | 0.003* | *F*(1, 183) = 4.15 | 0.043** |
|  | Task switching 1 | 81.87 (41.25) | 115.13 (61.15) | *F*(1, 199) = 7.53 | 0.007** | *F*(1, 180) = 2.22 | 0.138 |
|  | Task switching 2 | 48.19 (34.79) | 71.37 (55.99) | *F*(1, 199) = 4.27 | 0.040** | *F*(1, 180) = 0.86 | 0.354 |
| Psychomotor speed | Psychomotor speed 1 | 34.31 (16.21) | 45.03 (19.58) | *F*(1, 207) = 5.02 | 0.026** | *F*(1, 187) = 1.86 | 0.174 |
|  | Psychomotor speed 2 | 57.38 (18.91) | 46.41 (14.91) | *F*(1, 191) = 6.04 | 0.015** | *F*(1, 171) = 0.76 | 0.383 |
|  |  |  |  |  |  |  |  |
|  |  | **Controls** | | | | | |
| **Feeling of not being loved** |  |  |  |  |  |  |  |
| Learning and memory | Verbal learning | 59.36 (9.09) | 55.09 (11.27) | *F*(1, 190) = 2.70 | 0.102 | *F*(1, 181) = 1.67 | 0.198 |
|  | Consolidation | 1.01 (1.50) | 2.64 (4.13) | *F*(1, 191) = 8.49 | 0.004* | *F*(1, 185) = 51.72 | < 0.001* |
|  | Long-term memory | 1.17 (2,18) | 2.91 (3.67) | *F*(1, 189) = 5.78 | 0.017** | *F*(1, 183) = 22.52 | < 0.001* |
|  | Recognition | 13.24 (2.99) | 12.70 (3.16) | *F*(1, 188) = 0.17 | 0.680 | *F*(1, 182) = 0.31 | 0.580 |
|  | Short-term memory | 10.86 (2.27) | 10.91 (2.21) | *F*(1, 194) = 0.13 | 0.721 | *F*(1, 188) = 0.07 | 0.787 |
| Executive function | Working memory | 8.03 (2.52) | 7.91 (2.12) | *F*(1, 193) = 0.01 | 0.942 | *F*(1, 187) = 0.00 | 0.994 |
|  | Task switching 1 | 53.90 (25.61) | 55.45 (23.51) | *F*(1, 193) = 0.05 | 0.826 | *F*(1, 182) = 0.08 | 0.771 |
|  | Task switching 2 | 29.96 (19.96) | 27.91 (21.05) | *F*(1, 189) = 1.12 | 0.292 | *F*(1, 180) = 0.06 | 0.801 |
| Psychomotor speed | Psychomotor speed 1 | 23.98 (10.70) | 27.55 (9.49) | *F*(1, 193) = 1.33 | 0.251 | *F*(1, 187) = 0.03 | 0.876 |
|  | Psychomotor speed 2 | 85.95 (18.46) | 84.64 (14.39) | *F*(1, 190) = 0.07 | 0.707 | *F*(1, 181) = 0.29 | 0.588 |
| **Physical abuse** |  |  |  |  |  |  |  |
| Learning and memory | Verbal learning | 59.95 (8.73) | 50.89 (10.41) | *F*(1, 190) = 8.51 | 0.004* | *F*(1, 181) = 2.36 | 0.126 |
|  | Consolidation | 1.00 (1.47) | 2.11 (3.50) | *F*(1, 191) = 3.67 | 0.057 | *F*(1, 185) = 17.58 | < 0.001* |
|  | Long-term memory | 1.13 (2.15) | 2.67 (3.27) | *F*(1, 189) = 6.50 | 0.012** | *F*(1, 183) = 8.76 | 0.003* |
|  | Recognition | 13.36 (2.97) | 11.83 (3.00) | *F*(1, 188) = 0.96 | 0.330 | *F*(1, 182) = 0.02 | 0.882 |
|  | Short-term memory | 8.21 (2.48) | 9.00 (2.52) | *F*(1, 194) = 6.59 | 0.011** | *F*(1, 188) = 3.06 | 0.082 |
| Executive function | Working memory | 8.21 (2.48) | 6.22 (1.80) | *F*(1, 190) = 5.07 | 0.026** | *F*(1, 187) = 1.71 | 0.192 |
|  | Task switching 1 | 52.04 (23.35) | 73.17 (36.30) | *F*(1, 191) = 1.21 | 0.272 | *F*(1, 182) = 0.35 | 0.552 |
|  | Task switching 2 | 11.05 (2.16) | 43.00 (30.35) | *F*(1, 189) = 0.61 | 0.434 | *F*(1, 180) = 0.66 | 0.418 |
| Psychomotor speed | Psychomotor speed 1 | 23.58 (10.38) | 30.17 (11.66) | *F*(1, 193) = 0.60 | 0.441 | *F*(1, 187) = 0.34 | 0.563 |
|  | Psychomotor speed 2 | 87.49 (17.87) | 70.00 (13.89) | *F*(1, 190) = 5.72 | 0.018** | *F*(1, 181) = 3.43 | 0.066 |
| **Feeling of being hated** |  |  |  |  |  |  |  |
| Learning and memory | Verbal learning | 59.48 (9.21) | 55.58 (9.31) | *F*(1, 189) = 3.37 | 0.068 | *F*(1, 181) = 4.84 | 0.029** |
|  | Consolidation | 1.04 (1.51) | 1.68 (3.40) | *F*(1, 190) = 2.04 | 0.155 | *F*(1, 184) = 12.56 | < 0.001* |
|  | Long-term memory | 1.19 (2.18) | 2.11 (3.23) | *F*(1, 188) = 2.61 | 0.108 | *F*(1, 182) = 10.79 | 0.001* |
|  | Recognition | 13.26 (3.04) | 12.84 (2.69) | *F*(1, 187) = 0.19 | 0.663 | *F*(1, 181) = 0.19 | 0.667 |
|  | Short-term memory | 10.87 (2.27) | 10.80 (2.33) | *F*(1, 193) = 0.00 | 0.953 | *F*(1, 187) = 0.00 | 0.999 |
| Executive function | Working memory | 8.10 (2.50) | 7.60 (2.33) | *F*(1, 192) = 0.66 | 0.416 | *F*(1, 187) = 0.10 | 0.758 |
|  | Task switching 1 | 53.21 (24.33) | 61.40 (33.91) | *F*(1, 190) = 0.90 | 0.345 | *F*(1, 182) = 1.94 | 0.164 |
|  | Task switching 2 | 29.18 (18.54) | 35.70 (29.77) | *F*(1, 189) = 0.08 | 0.774 | *F*(1, 180) = 2.59 | 0.110 |
| Psychomotor speed | Psychomotor speed 1 | 24.01 (10.73) | 25.70 (10.02) | *F*(1, 193) = 0.82 | 0.336 | *F*(1, 187) = 0.00 | 0.971 |
|  | Psychomotor speed 2 | 86.23 (18.37) | 82.50 (17.48) | *F*(1, 189) = 0.94 | 0.334 | *F*(1, 181) = 1.14 | 0.288 |
| **Sexual abuse** |  |  |  |  |  |  |  |
| Learning and memory | Verbal learning | 59.18 (9.39) | 58.37 (8.24) | *F*(1, 189) = 0.55 | 0.460 | *F*(1, 181) = 0.54 | 0.463 |
|  | Consolidation | 1.13 (1.80) | 0.84 (1.61) | *F*(1, 190) = 1.33 | 0.251 | *F*(1, 184) = 4.13 | 0.044** |
|  | Long-term memory | 1.30 (2.40) | 1.05 (1.31) | *F*(1, 188) = 0.39 | 0.535 | *F*(1, 182) = 0.88 | 0.349 |
|  | Recognition | 13.18 (3.10) | 13.58 (1.95) | *F*(1, 187) = 1.79 | 0.182 | *F*(1, 181) = 1.19 | 0.278 |
|  | Short-term memory | 10.97 (2.24) | 9.89 (2.36) | *F*(1, 193) = 1.47 | 0.227 | *F*(1, 187) = 0.99 | 0.320 |
| Executive function | Working memory | 8.11 (2.53) | 7.39 (1.82) | *F*(1, 192) = 0.31 | 0.581 | *F*(1, 187) = 0.16 | 0.691 |
|  | Task switching 1 | 53.47 (25.92) | 59.42 (20.89) | *F*(1, 190) = 1.18 | 0.279 | *F*(1, 182) = 0.41 | 0.524 |
|  | Task switching 2 | 29.64 (20.38) | 31.79 (16.02) | *F*(1, 189) = 0.69 | 0.407 | *F*(1, 180) = 0.81 | 0.371 |
| Psychomotor speed | Psychomotor speed 1 | 23.81 (10.55) | 27.63 (11.17) | *F*(1, 193) = 0.58 | 0.590 | *F*(1, 187) = 0.66 | 0.416 |
|  | Psychomotor speed 2 | 86.13 (18.64) | 83.26 (14.61) | *F*(1, 189) = 0.56 | 0.454 | *F*(1, 181) = 0.10 | 0.751 |
| **Neglect** |  |  |  |  |  |  |  |
| Learning and memory | Verbal learning | 59.59 (8.94) | 46.57 (9.09) | *F*(1, 190) = 6.97 | 0.009** | *F*(1, 182) = 2.46 | 0.118 |
|  | Consolidation | 1.08 (1.77) | 1.71 (1.98) | *F*(1, 191) = 0.37 | 0.541 | *F*(1, 185) = 0.31 | 0.580 |
|  | Long-term memory | 1.22 (2.30) | 2.57 (2.23) | *F*(1, 189) = 1.98 | 0.161 | *F*(1, 183) = 0.86 | 0.354 |
|  | Recognition | 13.29 (2.93) | 11.14 (4.10) | *F*(1, 188) = 2.02 | 0.157 | *F*(1, 182) = 0.24 | 0.240 |
|  | Short-term memory | 10.89 (2.26) | 10.41 (2.55) | *F*(1, 194) = 0.06 | 0.801 | *F*(1, 188) = 0.37 | 0.767 |
| Executive function | Working memory | 8.06 (2.50) | 7.00 (2.16) | *F*(1, 193) = 0.37 | 0.543 | *F*(1, 187) = 0.00 | 0.960 |
|  | Task switching 1 | 52.68 (24.24) | 89.14 (6.75) | *F*(1, 191) = 6.87 | 0.009** | *F*(1, 182) = 1.66 | 0.199 |
|  | Task switching 2 | 28.73 (18.77) | 59.71 (29.04) | *F*(1, 189) = 7.62 | 0.006** | *F*(1, 180) = 1.82 | 0.179 |
| Psychomotor speed | Psychomotor speed 1 | 23.99 (10.72) | 29.43 (6.75) | *F*(1, 193) = 0.95 | 0.331 | *F*(1, 187) = 0.19 | 0.661 |
|  | Psychomotor speed 2 | 86.48 (18.13) | 69.57 (13.34) | *F*(1, 190) = 0.46 | 0.498 | *F*(1, 181) = 0.04 | 0.841 |
| * p < 0.005 significant (corrected for multiple testing)  ** p < 0.05 numerical, not significant differences  Model 1: adjusted for age and sex (if necessary)  Model 2: adjusted for age sex (if necessary), educational level, treatment setting and Positive and Negative Syndrome Scale  **^†^**Verbal learning, sum of correct words in rounds 1 to 5 of VLMT (words); consolidation, immediate loss of recalled words in VLMT (words); long-term memory, loss of recalled words after 25 minutes in VLMT (words); recognition, recognition of words in VLMT (words); short-term memory, Digit Span forward (correct numbers); working memory, Digit Span backward (correct numbers); task switching 1, TMT B (seconds); task switching 2, TMT B - TMT A (seconds); psychomotor speed 1, TMT A (seconds); psychomotor speed 2, Digit Symbol Test (symbols)  ELS, participants with early life stress; no ELS, participants reporting no significant early life stress; SD, standard deviation; TMT, Trail Making Test; VLMT, Verbal Learning and Memory Test | | | | | | | |
