# Supplementary material for: Association of early life stress and cognitive performance in patients with schizophrenia and healthy controls
Source: Schizophr Res Cogn. 2023 Feb 11;32:100280. doi: 10.1016/j.scog.2023.100280 (PMC9945796; doi:10.1016/j.scog.2023.100280)
Supplement: Supplementary file 2 — Supplementary material [file mmc2.docx]

**Supplementary Material.**

### Trail Making Test

The Trail Making Test (TMT) measures multiple cognitive domains, including visual attention (Parts A and B), psychomotor speed (Part A), and task switching (Part B), and provides a good estimate of executive function. Participants are asked to connect digits in increasing order (Part A) or digits in increasing order and letters alphabetically alternately (Part B). The time needed for each part of the test is measured, and the time taken to switch between tasks is estimated by subtracting the time for Part A from the time for Part B (Tischler and Petermann, 2010).

### Verbal Digit Span

The Verbal Digit Span (VDS) test assesses short-term (forward digit span) and working memory (backward digit span). Participants are asked to repeat seven pairs of number sequences with increasing length, either forwards, or backwards, receiving a point score for each correctly repeated sequence. One point is given for each correctly recalled string of digits, and the interviewer proceeds until the participant makes mistakes in recalling strings of the same length. All points are summed to obtain the final score (Herbstrith, 2016).

### Digit Symbol Test

The Digit Symbol Test (DST) measures psychomotor speed. Participants are presented with rows of numbers with a space below each number. Each number is associated with a symbol, and the number-symbol key is given at the top of the test sheet. Participants are instructed to match each number with the respective symbol and fill in as many symbols as possible in 120 seconds. The final score is the total number of correct symbols (Molz et al., 2010) .

### Verbal Learning Memory Test

The Verbal Learning Memory Test (VLMT) evaluates verbal learning and memory and is the German version of the Rey Auditory Verbal Learning Test (Bean, 2011). Fifteen words are verbally presented to participants five times, and participants are asked to repeat the words after each presentation. Next, a different list of words is presented once, and participants are asked to repeat this list. Immediately and 25 to 30 minutes later, participants are asked to recall the words from the first list. Finally, interviewers test recognition of the words from the first list by verbally presenting 50 words from both lists and semantically or phonetically similar new words and asking participants to indicate which words were in the first list. Four VLMT scores are calculated representing four cognitive subdomains: (1) total number of correctly recalled words in the five presentations of the first list (verbal learning); (2) words from the first list that were lost after participants were distracted by the reading of the second list (consolidation); (3) words that were lost after 25 to 30 minutes (long-term memory); and (4) correctly recognized words in the list of 50 words (recognition). Higher scores for consolidation and long-term memory indicate worse performance, and higher scores for verbal learning and recognition, better performance.

**References**

Bean, J., 2011. Rey Auditory Verbal Learning Test, Rey AVLT, in: Kreutzer, J.S., DeLuca, J., Caplan, B. (Eds.), Encyclopedia of Clinical Neuropsychology. Springer, New York, NY, pp. 2174–2175. https://doi.org/10.1007/978-0-387-79948-3_1153

Helmstaedter, Christoph, Lendt, Michael, Lux, Silke, 2001. Verbaler Lern- und Merkfähigkeitstest: VLMT ; Manual. Beltz-Test.

Herbstrith, J.C., 2016. Book Review: *WISC-V assessment and interpretation: Scientist-practitioner perspectives* By Weiss, L. G., Saklofske, D. H., & Holdnack, J. A. (Eds.). J. Psychoeduc. Assess. 34, 97–100. https://doi.org/10.1177/0734282915615449

Tischler, L., Petermann, F., 2010. Trail Making Test (TMT). Z. Für Psychiatr. Psychol. Psychother. 58, 79–81. https://doi.org/10.1024/1661-4747.a000009
